# Acute lymphocytic myocarditis characterized by cardiogenic shock and conduction system abnormalities in patients with Hashimoto’s thyroiditis: a case report and review of literature

**DOI:** 10.1093/ehjcr/ytae268

**Published:** 2024-06-11

**Authors:** Germana Panattoni, Marco Marino, Andrea Ascione, Giulia d’Amati, Leonardo Calò

**Affiliations:** Division of Cardiology, Policlinico Casilino, Via Casilina 1049, 00169 Rome, Italy; Division of Cardiology, ‘Tor Vergata’ University Hospital, Rome, Italy; Department of Experimental Medicine, Sapienza University of Rome, Rome, Italy; Department of Radiological, Oncological and Pathological Sciences, Sapienza University of Rome, Rome, Italy; Division of Cardiology, Policlinico Casilino, Via Casilina 1049, 00169 Rome, Italy

**Keywords:** Case report, Acute myocarditis, Cardiogenic shock, Hashimoto’s thyroiditis, Conduction system abnormalities

## Abstract

**Background:**

Acute myocarditis (AM) is an inflammatory heart disease that may occur as a consequence of autoimmune disorders. Although the correlation between myocarditis and hyperthyroidism has been reported in the literature, the association with hypothyroidism is less frequent.

**Case summary:**

We describe a characteristic case of lymphocytic acute myocarditis deteriorated into cardiogenic shock due to Hashimoto’s thyroiditis treated with vasopressor and inotropic drugs in combination with corticosteroid. On admission, electrocardiography revealed a sinus tachycardia with 1st degree atrioventricular (AV) block, right bundle branch block (RBBB), and left anterior fascicular block. Laboratory tests demonstrated a severe hypothyroidism and high-titre serum of antibodies against thyroglobulin. She presented a favourable clinical course, restoring haemodynamic stability. A resolution of hypothyroidism and a progressive reduction of the value of antibodies against thyroglobulin occurred. On Day 35, the patient was discharged showing on electrocardiogram the occurrence of left posterior fascicular block, disappearance of 1st degree AV block and partial improvement of RBBB along with the normalization of the left ventricular contractility abnormalities on echocardiography.

**Discussion:**

Autoimmune features, mostly Hashimoto’s thyroiditis, are associated in lymphocytic acute myocarditis to a worse prognosis and an increased risk of recurrence. More studies are needed to elucidate the underlying mechanism.

**Learning points**

- Cardiogenic shock is a potential form of clinical presentation of acute lymphocytic myocarditis. Referral to centre expertise is necessary in order to consider a prompt treatment.
- New-onset conduction system disease could be related to inflammatory infiltration of the cardiac conduction system in acute lymphocytic myocarditis.
- Autoimmune features, mostly Hashimoto’s thyroiditis, are associated in lymphocytic acute myocarditis to a worse prognosis and an increased risk of recurrence.

## Introduction

Acute myocarditis (AM) is an inflammatory heart disease, diagnosed by established histological, immunological, and immunohistochemical criteria, that may occur as consequence of infections, exposure to toxic substances, drugs, and immune system activation.^1^ Based on the cell types infiltrating, myocarditis can be classified as eosinophilic, lymphocytic, giant cells, or granulomatous.

The presentation pattern can range from non-specific symptoms such as chest pain associated with fatigue and shortness of breath to more aggressive features characterized by left ventricular systolic dysfunction, ventricular arrhythmias (VA), or cardiogenic shock. Life-threatening VA may present at any stage of the disease as an expression of myocardial electrical instability. Brady-arrhythmias are infrequent except for some special aetiologies (cardiac sarcoidosis, giant cell myocarditis, Chagas disease, or some systemic autoimmune diseases). Acute myocarditis can be associated with autoimmune disorders or organ/system-specific autoimmune/inflammatory diseases.^1–3^

In this setting, although the correlation between myocarditis and hyperthyroidism is well documented in the literature,^4^ association with hypothyroidism remains less understood. We describe a characteristic case of AM associated with severe hypothyroidism due to Hashimoto’s disease.

## Summary figure

Ab, antibodies; ANA, antinuclear antibodies; anti-TG, anti-thyroglobulin; CMR, cardiac magnetic resonance; EMB, endomyocardial biopsy; ECG, electrocardiogram; Echo, echocardiography; LAFB, left anterior fascicular block; LGE, late gadolinium enhancement; LPFB, left posterior fascicular block; LVEF, left ventricular ejection function; RBBB, right bundle branch block; TPO, antithyroid peroxidase.

**Figure:**
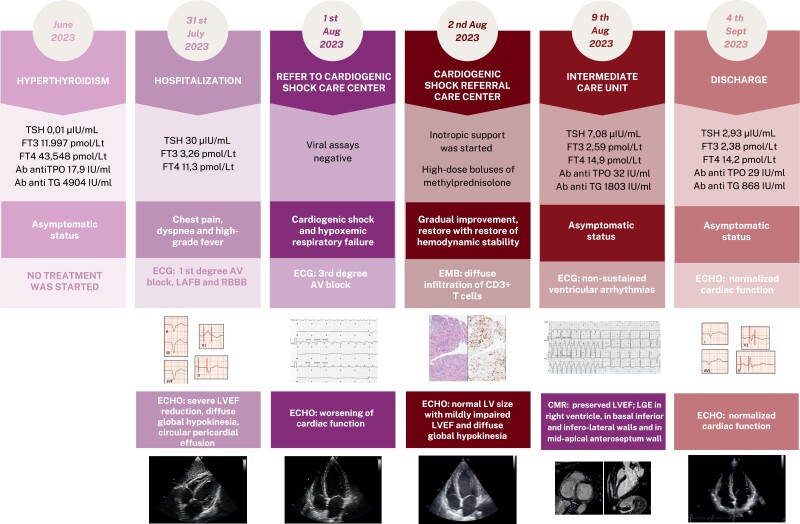


## Case presentation

A 49-year-old woman with no family history of cardiovascular disease and no cardiovascular risk factors was admitted to the Emergency Department of our hospital with persistence of high-grade fever (up to 39.0°C), dyspnoea and sub-sternal oppressive chest pain, which worsened during inspiration and improved by sitting up and leaning forward. Her medical history reported a previous diagnosis of hyperthyroidism three months before hospitalization but no medical treatment was started.

**Figure 1:**
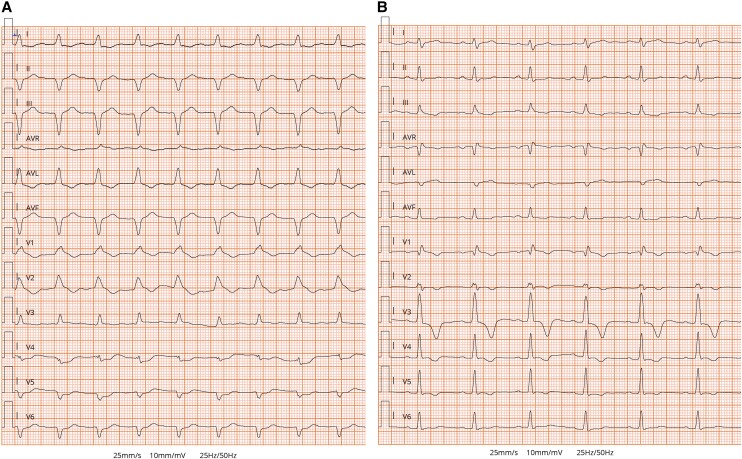
Electrocardiogram. (*A*) Initial ECG on arrival to emergency department showing sinus tachycardia with 1st degree atrioventricular (AV) block, complete right bundle branch block (RBBB), and left anterior fascicular block (LAFB). (*B*) The ECG performed on discharge showed resolution of 1st degree AV block and LAFB and the occurrence of left posterior fascicular block (LPFB) and partial improvement of RBBB.

On admission, general examination was unremarkable. Blood pressure was 110/70 mmHg, and pulse rate was 110 beats per min. Her body temperature was 37.8°C. Electrocardiogram (ECG) revealed sinus tachycardia with 1st degree atrioventricular (AV) block, right bundle branch block (RBBB), and left anterior fascicular block (LAFB) (*Figure 1A*). Bedside transthoracic echocardiography (TTE) showed a normal left ventricular (LV) size with severely impaired cardiac function (LV ejection fraction ∼20%) and diffuse global hypokinesia. Right ventricular systolic function was mildly impaired with a tricuspid annular plane systolic excursion of 13 mm and circular pericardial effusion (see Supplementary material online, *Video 1*). Emergency coronary angiography documented non-obstructive coronary artery disease.

The patient was admitted to the coronary care unit, in stable general conditions; no oxygenation disorder was observed. Laboratory tests showed high sensitivity cardiac Troponin T peak at 3202 pg/mL and a significant hypothyroidism (TSH 30 µIU/mL, FT3 3.26 pmol/L, FT4 11.3 pmol/L). Levothyroxine replacement therapy was started (25 µg). Results of viral assays (Herpes Virus, Epstein–Barr virus, Parvovirus, Cytomegalovirus, and Coxsackie) were negative, and the rheumatologic panel demonstrated the presence of antinuclear antibodies (ANA) and anti-DNA antibodies.

On Day 2, the patient developed pulmonary oedema with hypoxemic respiratory failure, requiring loop diuretics and mechanical ventilation; she quickly deteriorated into cardiogenic shock characterized by hypotension and clinical (cold sweated extremities, oliguria, mental confusion, dizziness) and biochemical (metabolic acidosis and elevated serum lactate) manifestations of hypoperfusion. An inotropic support was initiated. She developed a 3rd degree AV block. Despite supportive measures, her clinical status continued to deteriorate. Therefore, the patient was transferred to the cardiogenic shock referral care centre.

**Figure 2:**
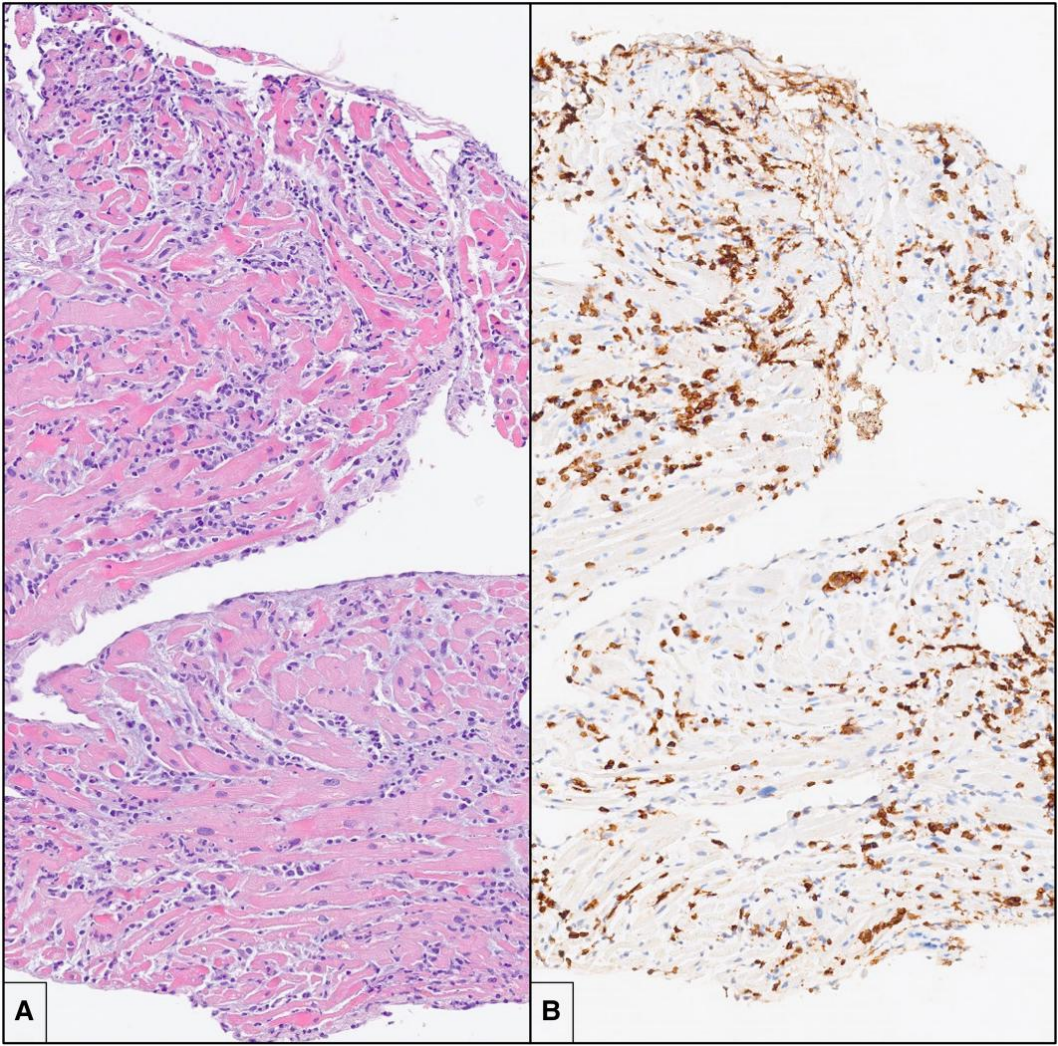
Endomyocardial biopsy. (*A*) There is an intense and diffuse mononuclear interstitial inflammatory infiltrate in association with damage and necrosis of cardiac myocytes (haematoxylin and eosin; original magnification: 10×). (*B*) Immunohistochemistry for CD3 shows that a large portion of the inflammatory infiltrate is composed by CD3+ T lymphocytes, with formation of clusters (immunohistochemistry, original magnification: 10×).

Inotropic support with epinephrine (0.05 mg/kg/min) and dobutamine (5 mg/kg/min) was started, and levosimendan (0.1 mg/kg/min) was administered with a significant progressive improvement in cardiac function. On Day 3, endomyocardial biopsy was performed: histology showed intense mononuclear inflammatory interstitial pattern with diffused cardiomyocyte damage. On immunohistochemistry, most inflammatory cells were CD3+ T lymphocytes and CD68+ macrophages (*Figure 2*). A diagnosis of severe, active, lymphocytic myocarditis was formulated. The search for viral genomes on sampled tissue and blood was negative. The patient received systemic loop diuretics and high-dose boluses of methylprednisolone (1 g/day during 72 h) under suspicion of AM virus negative. She presented a favourable clinical course, restoring haemodynamic stability. After the gradual weaning of inotropic support, on Day 4, extubation was planned. On Day 8, TTE revealed a normal LV size with mildly impaired cardiac function (LV ejection function 45%) and diffuse global hypokinesia.

**Figure 3:**
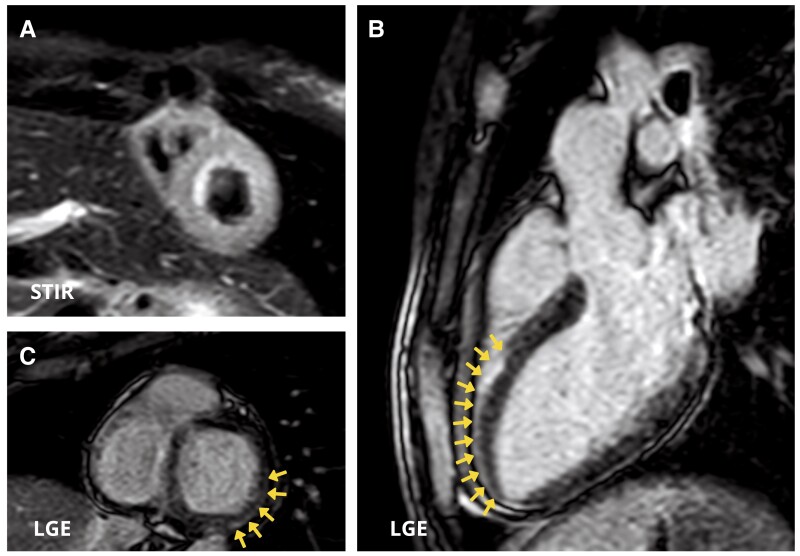
Cardiac magnetic resonance images. (*A*) Short-axis view showing diffuse oedema in the left ventricular wall in T2 Short Time inversion recovery (STIR) sequences. (*B* and *C*) Three-chamber view (*B*) and short-axis view (*B*) demonstrate myocardial late gadolinium enhancement with sub-epicardial distribution in the mid-apical segment of antero-septal wall and in the basal segment of inferior and infero-lateral walls (arrows).

**Figure 4:**
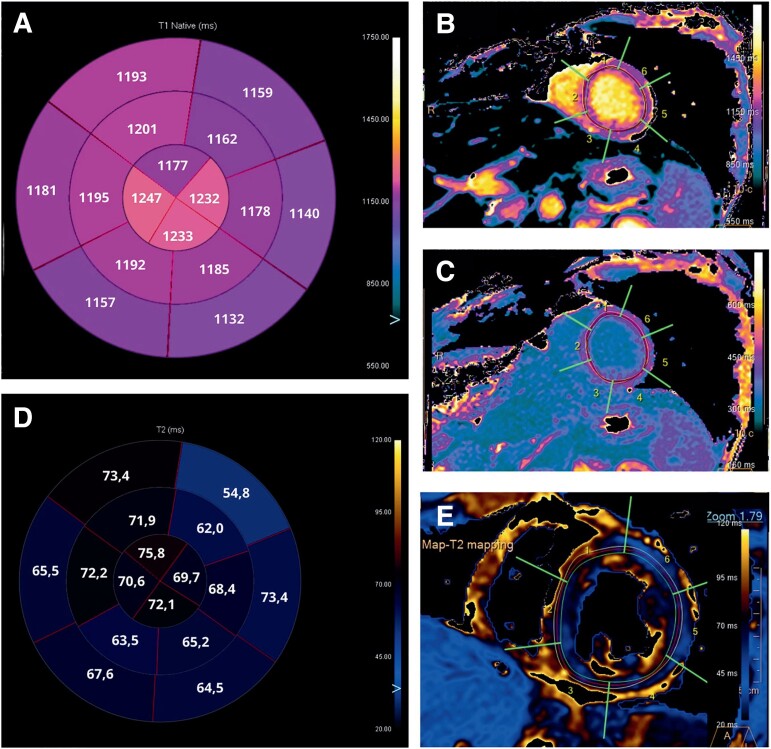
Cardiac magnetic resonance images. (*A*) Bullseye plot showing the native T1 times. Segmentation was performed according to the AHA 16-segment model. (*B* and *C*) Native T1 times and T1-enhanced were increased in all segments, especially in mid and apical segments. (*D*) Bullseye plot showing the native T2 times. (*E*) Native T2 times were increased in all segments, especially in mid and apical segments.

The patient was transferred to the intermediate care unit (ICU) on Day 10. Laboratory tests demonstrated TSH 7,08 µIU/mL, FT3 2,59 pmol/L, FT4 14,9 pmol/L, antibodies against thyroid peroxidase 32 IU/mL (normal value < 60 IU/mL), and thyroglobulin 1803 IU/mL (normal value < 115 IU/mL). Electrocardiogram, performed on Day 10, reported an improvement in conduction abnormalities, marked by the resolution of 1st degree AV block and LAFB with the occurrence of the left posterior fascicular block and partial improvement of RBBB (*Figure 1B*) along with the normalization of LV systolic function (see Supplementary material online, *Video 2*). Electrocardiogram monitoring revealed non-sustained VA. Cardiac magnetic resonance with gadolinium contrast, including T1 and T2 mapping and T2-STIR sequences, was performed on Day 12 using a 1.5 T scanner (*Figures 3* and *4*) (see Supplementary material online, *Video 3*). Corticosteroid (prednisone 1 mg/kg daily) therapy was administered for 4 weeks, followed by 0.33 mg/kg for 5 months. Additionally, azathioprine 2 mg/day was initiated as a maintenance agent. During hospitalization in ICU, she remained asymptomatic and on Day 35, she was transferred to a rehabilitation centre.

## Discussion

We are reporting a characteristic case of AM associated with severe hypothyroidism due to Hashimoto’s disease. As known, AM can be linked to autoimmune disorders. Identifying the myocarditis-related condition is crucial for specific therapies. The presentation pattern can range from non-specific symptoms to a more aggressive features characterized by cardiogenic shock.^2^ In our experience, early allocation to a centre with expertise in cardiac assist devices was necessary in order to consider a prompt and specific treatment if conservative pharmacologic approaches had failed.^3^

Brady-arrhythmias, occurred in our patient, are infrequent, except for some special aetiologies or some systemic autoimmune diseases involving the myocardium. The pathophysiology of heart blocks has been attributed to a selective infiltration by CD3+ T mononuclear cells of the conduction system.^5,6^ The same electrocardiographic alterations, reported in our patient, were described in a 53-year-old man with AM, who died due to multiorgan failure. In our experience, the progressive resolution of inflammation and extensive injury resulted in LAFB disappearance, resolution of AV block, and an incomplete RBBB appearance.

Endomyocardial biopsy confirmed the diagnosis of lymphocytic myocarditis^1,3^ and influenced the treatments and prognosis, since it was the basis for safe (infection-negative) immunosuppression treatment.^7,8^ Cardiac magnetic resonance imaging provides non-invasive tissue characterization of the myocardium, according to Lake-Louise criteria.^9^

The immunological profile of our patient revealed a high-titre serum of antibodies against thyroglobulin, DNA, and ANA in a clinical setting compatible with Hashimoto’s thyroiditis. Hashimoto’s thyroiditis is the most frequent autoimmune thyroid disorder and the leading cause of hypothyroidism. In patients with autoimmune thyroid disorders, it is common to report a high prevalence of antibodies against DNA, since ∼20% of patients is suffering from other organ-specific/systemic autoimmune diseases. The association is rooted in both common genetic susceptibility and environmental factors.^10^ In these disorders, there is a common immune-pathogenic pathway, in which Th1-orientated immune response prevails. Lymphocytic infiltration, especially of T cells, and follicular destruction are the histological hallmarks of autoimmune thyroiditis, leading to gradual atrophy and fibrosis. However, few data are available on the association between thyroid disease and lymphocytic AM. Although the pathogenic mechanisms of the associations are not clear, we can assume that immune defects, immunological cross-reactivity, hormones, and genetic and environmental factors may play a central role in poly-autoimmunity.^10^ Autoimmune features, mostly Hashimoto’s thyroiditis, are associated in lymphocytic AM to a worse prognosis and an increased risk of recurrence, as reported after 20-year follow-up of the TIMIC trial.^7^

*Table 1* ^11–16^ summarizes published reports and case series of autoimmune thyroid disorders associated with lymphocytic myocarditis. Lorin De La Grandmaison *et al*.^11^ reported an autopsy case of lymphocytic myocarditis associated with Hashimoto’s thyroiditis: in both the heart and thyroid gland had a nodular pattern with germinal centre with prevalence of CD3+ and CD5+ T lymphocytes.

**Table 1:** Summary of principal studies reporting lymphocytic myocarditis or heart failure as presenting onset of autoimmune thyroid diseases

Studies	Type	Thyroid function	Antibodies	Diagnosis	Presentation	CRM	Coronary angiography	Endomyocardial biopsy
Lorin De La Grandmaison G *et al*.^11^	CR	Hypothyroidism Hashimoto’s thyroiditis	Anti-thyroglobulin antibodies Antithyroid peroxidase antibody	Hashimoto’s thyroiditis	Sudden cardiac death	Not performed	Not performed	Lymphocytic infiltration (T CD3-CD5) with same macrophages)
Mavrogeni *et al*.^12^	CS	Early phase of hyperthyroidism, then normal thyroid function	Anti-microsomal and anti-thyroglobulin antibodies	Not available	Chest pain Dyspnoea Palpitations	Positive 15/50 patients. LGE was identified in infero-lateral wall of 8/15.	Normal coronaries	Lymphocytic myocarditis patter
Fatourechi and Edwards^13^	CS	Hyperthyroidism Hypothyroidism	Not available	59 patients Graves’ disease 49 patients Hashimoto’s thyroiditis	Low-output heart failure	Not performed	Not performed	11 biopsy available: 2 (18%) showed lymphocytic infiltrates
Chen *et al*.^14^	CR	Hyperthyroidism	Antithyroid peroxidase antibody Anti-TSH receptor antibody	Graves’ disease with Hashimoto autoimmune component	Hyperpyrexia and sinus tachycardia evolved in low-output heart failure	Not performed	Not performed	Lymphocytic myocarditis with moderate T lymphocyte
Demoulin *et al*.^15^	CR	Hypothyroidism	Anti-thyroglobulin antibody Antithyroid peroxidase antibodies	Hashimoto’s thyroiditis	Sudden cardiac death	Not performed	Not performed	Lymphocytic infiltration (T CD3 and T CD5 associated with B CD20 lymphocytes) and some macrophages
Yagoro *et al*.^16^	CR	Hypothyroidism	Anti-microsomal antibodies	Post-partum autoimmune thyroiditis	Syncope AV block	Not performed	Normal coronaries	Mild lymphocytic infiltration and disarrangement of myocytes

Finally, more studies are needed to elucidate the true prevalence of lymphocytic myocarditis related to autoimmune thyroiditis and clarify a possible common underlying autoimmune mechanism.

## Supplementary Material

ytae268_Supplementary_Data

## Data Availability

The data underlying this article will be shared on reasonable request to the corresponding author.

## Lead author biography

Dr Germana Panattoni received her medical degree at the University of Rome, Tor Vergata, Italy in 2018. She obtained her Speciality Training in Cardiology in 2016 and earned a PhD in Systems and Experimental Medicine in 2020 at the University of Rome, Tor Vergata, Italy. She is currently working as a Cardiologist at Policlinico Casilino, Rome. Her main areas of interest are cardiac arrhythmias and intensive cardiac care.

## Supplementary material

**Consent:** The authors confirm that written consent for submission and publication of this case report including images and associated text has been obtained from the patient in line with COPE guidance.

**Funding:** None declared.
